# Global Health Philanthropy and Institutional Relationships: How Should Conflicts of Interest Be Addressed?

**DOI:** 10.1371/journal.pmed.1001020

**Published:** 2011-04-12

**Authors:** David Stuckler, Sanjay Basu, Martin McKee

**Affiliations:** 1 Department of Global Health and Population, Harvard University, Boston, Massachusetts, United States of America; 2 Department of Public Health and Policy, London School of Hygiene & Tropical Medicine, London, United Kingdom; 3 Department of Medicine, University of California San Francisco, San Francisco, California, United States of America; 4 Division of General Internal Medicine, San Francisco General Hospital, San Francisco, California, United States of America

## Abstract

David Stuckler and colleagues examine five large private global health foundations and report on the scope of relationships between these tax-exempt foundations and for-profit corporations including major food and pharmaceutical companies.

**Summary Points**

- Institutional relationships in global health are a growing area of study, but few if any previous analyses have examined private foundations.
- Tax-exempt private foundations and for-profit corporations have increasingly engaged in relationships that can influence global health.
- Using a case study of five of the largest private global health foundations, we identify the scope of relationships between tax-exempt foundations and for-profit corporations.
- Many public health foundations have associations with private food and pharmaceutical corporations. In some instances, these corporations directly benefit from foundation grants, and foundations in turn are invested in the corporations to which they award these grants.
- Personnel move between food and drug industries and public health foundations. Foundation board members and decision-makers also sit on the boards of some for-profit corporations benefitting from their grants.
- While private foundations adopt standard disclosure protocols for employees to mitigate potential conflicts of interests, these do not always apply to the overall endowment investments of the foundations or to board membership appointments. The extent and range of relationships between tax-exempt foundations and for-profit corporations suggest that transparency or grant-making recusal of employees alone may not be preventing potential conflicts of interests between global health programs and their financing.

## Institutional Relationships and Global Health

The institutional relationships that exist in global health are a growing area of inquiry. This has been most evident in work examining corporate involvement in health, because tensions can arise between the profit motives of corporations and the promotion of public health. Whereas corporations make products that can improve health (such as pharmaceuticals and vaccines) and relationships between public health institutions and for-profit corporations can be seen as positive opportunities for corporations to improve public health [1], corporations also make products that damage health (such as tobacco or unhealthy foods). And because some corporations have a vested interest in the activities of public health bodies, there have been documented attempts to influence the public health agenda by establishing associations with health care institutions [2].

The relationships of concern between for-profit institutions and health-related organizations can involve direct financing—such as when pharmaceutical companies give gifts to physicians in a manner that has been shown to increase prescribing of the promoted drugs [3],[4]—or indirect relationship-building, such as when corporate staff or paid consultants act as board members or strategic advisors to health organizations. There are many documented examples of corporate tactics by the tobacco, mining, alcohol, asbestos, food, and petrochemical industries aimed at influencing public health promotion (Table 1) [5]–[7].

**Table 1:** Selected examples of corporate strategies to influence public health promotion.

Strategy	Selected Examples
Public relations	Emphasize consumer's personal responsibility, moderation, free choice, and pleasure.
	Use the “government” versus “personal freedom and civil liberties” and “get government off our backs” arguments.
	Vilify critics, health advocates and public health scientists as “health police” or “fascists” and accuse them of seeking to impose a “nanny state.”
	Hire a public relations firm to develop and help carry out plans to create a favorable image, combat negative reports, or repair damage to credibility or image.
	Set up or fund “front groups” with consumer advocacy sounding names to promote the corporate agenda and messages.
Distortion of science	Divert attention from health effects of their product or practices to other matters.
	Publish journal articles and book chapters, make presentations at scientific meetings, host conferences and workshops for professionals that give the appearance of objective science in order to convey an image of credibility, but do not present the entire dataset, or misrepresent or distort data about the corporation's harmful operations, products or policies.
	Pay scientists or physicians or other professionals to serve as spokespersons to represent the corporation's position.
Political influence	Use lobbying to gain competitive advantage, avoid or minimize regulation and taxation.
	Contribute funds to election campaigns of politicians in positions to influence legislation favorable to the corporation and to obtain favorable rulings from the judiciary.
	Participate as delegates in the policy-making or standard setting process to ensure the lowest or most lenient possible standards for corporate products and operations.
	Work to reduce government budgets for scientific, policy, and regulatory activities deemed contrary to the corporation's profit.
Financial tactics	Contribute funding to community and neighborhood organizations in order to create dependency, gain allies, and influence or manipulate the organization's agenda.
	Set up or fund foundations that support the corporation's agenda rather than funding priorities determined through independent democratic processes (i.e., taxes).
	Externalize as much cost as possible (e.g., dumping chemicals into rivers; not providing employee medical coverage).
Legal and regulatory tactics	Work to get corporate officials or industry lobbyists appointed to governmental regulatory agencies with authority over its own industry.
	Shop for other judiciary venues or levels of government when rulings or decisions are unfavorable.
	In regulatory or judicial matters, avoid as long as possible having a hearing on the facts.
	When deemed necessary to ensure profits, employ illegal means.
Products and services	Emphasize technological solutions to health problems to generate profit.
	Use both direct advertising and indirect methods such as product placement and integration into the story line of entertainment venues.
	Connect image of product or corporation with human emotions and values.

Sources: Adapted from [6],[81].

In addition to examining corporate relationships in the health arena, public health advocates have also expressed concerns about whether potential conflicts of interests among state development aid programs could negatively affect health outcomes. Many government development agencies act explicitly in the interests of their own country [8]–[13]. This has taken the form of conditionalities on development aid and when aid is linked to other political agendas such as reduced tariffs or privatizing state-owned assets [14],[15]. Government aid has also been criticized for causing unintended health consequences—for example, by distorting recipient government health budgets by focusing on one or a few diseases at the expense of others [16], by creating a misalignment between health funding and the observed burden of disease due to external donations [17], and by inappropriately displacing government funds that would have otherwise been invested in health [18].

## Institutional Conflicts of Interests

When the agendas of institutions working in global health coincide, relationships can bring health benefits. But where agendas differ, a conflict of interest may arise. Sometimes termed “competing interests,” conflicts of interest have varying definitions. The strict, legal meaning of “conflicts of interest” under US law is a situation in which there is “a real or seeming incompatibility between one's private interests and one's public or fiduciary duties” [19]. In more popular usage, as set out in Wikipedia, it is defined as when “an individual or organization is involved in multiple interests, one of which could *possibly* corrupt the motivation for an act in the other [italics in original]” [20]. Disclosure is an essential first step in dealing with such potential conflicts [21], but in some cases these are fundamentally irreconcilable even with disclosure. It can also be difficult to ascertain when disclosure is only partial, as evidenced by tobacco litigation that provided examples of how it is possible to disclose some truth, but not the whole truth, when it came to declarations of conflicts of interest [22]. Thus, the World Health Organization (WHO) defines a potential conflict of interest more broadly, as in its guidelines for the Roll Back Malaria (RBM) program:

A conflict of interest can occur when a Partner's ability to exercise judgment in one role is impaired by his or her obligations in another role or by the existence of competing interests. Such situations create a risk of a tendency towards bias in favor of one interest over another or that the individual would not fulfill his or her duties impartially and in the best interest of the RBM Partnership.

A conflict of interest may exist even if no unethical or improper act results from it. It can create an appearance of impropriety that can undermine confidence in the individual, his/her constituency or organization. Both actual and perceived conflicts of interest can undermine the reputation and work of the Partnership. [23]

### Global Health Foundations and Potential Conflicts of Interest

While corporate involvement in and government aid for health has been extensively analyzed and critiqued in the public health literature, less attention has been paid to the impact of private donors on public health [24]. Over the past decade, the bulk of new health aid designed to reach the Millennium Development Goals has come from individuals and corporations [25],[26]. The influence of this private philanthropy on global health is profound and transformative [27].

Yet, it is intuitive that those who donate money have a set of goals and objectives that are of interest to them in making such donations. Although the philanthropic activities of wealthy individuals and corporations have attracted controversy in the past (Text S1), their charitable mission often means that they face less scrutiny than governments; critical analysis of foundations can be seen as “biting the hand that feeds us.” As a result, within the global health community, private donors are sometimes viewed as the “third rail” that no one wishes to analyze. However, by virtue of not being subject to popular elections, private foundations operate outside the typical boundaries of democracy; unlike government ministries, private foundations cannot be influenced in the same way by the communities affected by the foundations' actions. In the interests of public health, and particularly because poor communities affected by foundations do not automatically have a feedback mechanism to influence the decisions of private funders, we argue that it is appropriate to subject private foundations to the same scrutiny received by public institutions.

In this paper, we examine the scope of potential conflicts of interests that exist among the private foundations that are major funders of global health. We use the WHO definition of potential conflicts of interest, as detailed above. We conducted a case study using data about the networks, activities, and funding patterns of the Bill & Melinda Gates Foundation, Ford Foundation, W. K. Kellogg Foundation, Robert Wood Johnson Foundation, and Rockefeller Foundation, because these are the largest private global health foundations. We include an examination of both the potential conflicts that may arise in relation to these foundations' overall activities and those of their individual employees, which are already typically covered by the foundations' conflict of interest policies. We show that, taken together, these conflicts of interest can create complex situations whose potential effects may be difficult to assess in isolation.

Before proceeding with the findings of our analysis, we first review the governance and regulations of private nonprofit foundations.

### Rules and Regulations Governing Private Nonprofit Foundations

Nongovernmental, nonprofit organizations operate under different rules and regulations in each country. In the United States' common law system, private foundations must qualify for federal tax exemption. Unlike public charities, they usually do not provide services directly, but instead make grants to other entities to fulfill the foundation's mission. Text S2 provides detailed information about the rules and regulations governing private foundations in the US; most notably, private foundations must not operate in a manner that benefits private interests, particularly because their tax exemption implies that their work justifies forgoing the redistribution of tax dollars from their private accounts to public programs.

Private foundations may receive most of their funds from a parent for-profit corporation (such as PepsiCo Foundation) or wealthy individuals and families (such as the Doris Duke Foundation), sometimes in the form of investments (gifts of stock) and cash endowments. Historically some foundations have maintained separation between management of their endowments and grantmaking decisions [28], although these practices and the degree of separation vary across foundations. Some foundations, like the Ford Foundation, have investment committees composed of members of the board of directors. Others may manage foundation investments externally or as a collaboration of professional investment teams with members of the board. In most cases, the board of directors is responsible for overseeing that funding decisions are made according to a set of foundation grant-making criteria. Again, practices vary across foundations, as some have governance committees, but typically these criteria are set by the founders (in some cases including the parent company) and/or executive committee members, donors, and overall boards of directors of the foundations.

Despite the existence of policies explicitly designed to mitigate potential conflicts of interest, the boundaries between foundations, their investments, and their parent corporations or private funders can become blurred. Often, directors of the boards of foundations also sit on the board of private corporations, adopting multiple roles. Thus, the Hilton Foundation has “an independent Board of Trustees and Grants Committee comprised of independent as well as senior Hilton personnel. The Board of Trustees includes independent members and Hilton members nominated by the business” [29]. Although practices vary across foundations, typically the chief executive officer (CEO) of the parent corporation appoints the executives to the private foundation and has official authority to influence funding decisions through serving on the foundation board [30]. However, while acting as a member of the corporation's board, the CEO, as well as any other member of a corporate board, has a legally binding fiduciary duty to maximize shareholder profits for the corporation. For this reason, until 1953, US law prohibited companies from donating money to causes from which they did not directly benefit [30].

To understand whether measures currently employed to prevent potential conflicts of interest among private foundations are sufficient to prevent an adverse impact on public health, it is first necessary to evaluate the scope of these potential conflicts. Previous analyses of corporate boards using power structure methods have studied corporate philanthropy and economic development organizations [30],[31],[32],[33], but to our knowledge such analyses have yet to be applied to the area of public health [24],[27]. Here, we apply these methods for the first time to study major private funders of global health programs.

## Data and Methods

There are more than 100,000 private nonprofit foundations operating in the US, many of which contribute to global health activities and research (holding about US$569 billion in assets). Table 2 lists the top US-based private foundations. Of these, we chose the Bill & Melinda Gates Foundation as a focus for detailed case-study because it is the largest private philanthropy in global health, with financing greater than the budget of the entire WHO. For comparison, we chose the next three wealthiest US private grant-making foundations contributing to global health funding: the Ford Foundation, the W. K. Kellogg Foundation, and the Robert Wood Johnson Foundation. Given the historical importance of the Rockefeller Foundation in global health, we also included it as a fifth comparator. While our analysis is limited to these five foundations, the methods applied here can be extended to any other foundations and their institutional affiliates. Our intention is to provide an illustrative case study for initial analysis of this subject.

**Table 2:** Top five private foundations in the US, 2008.

Private Foundation	Revenue (US$)	Assets (US$)
Bill & Melinda Gates Foundation	3,760,987,546	29,673,548,843
Ford Foundation	817,196,296	11,045,127,839
J Paul Getty Trust	444,041,724	9,525,252,079
W K Kellogg Foundation	314,103,287	7,551,988,898
Robert Wood Johnson Foundation	1,809,718,672	7,505,353,587

Source of data: Urban Institute, based on IRS 990-PF reports. Available at: http://nccsdataweb.urban.org/PubApps/nonprofit-overview-segment.php?t=pf.

In the sociology and political science literature, aid organizations have typically been studied by examining at least three sets of observable questions about power and financial relationships, with the intent of “following the money” [33]–[39]: (i) Where does money come from and with what conditions?; (ii) Who decides? Who sits on the board of directors, and what are their histories, relationships, and interests?; and (iii) Who benefits from these decisions? Where are these funds distributed and which entities derive income from that expenditure? Related to decision-making and control of the agenda, notions of “soft power”[40] and Lukes' third dimension of power [41] emphasize the importance of power expressed not only through concrete, observable decisions and financial flows but also in hidden and more subtle, yet powerful, influences such as through shaping perceptions, cognition, and preferences, and influencing what is “kept out” of agendas. This dimension of power is arguably the most powerful; it is ideological in nature and addresses how decisions are made by the foundation about what issues are funded and how (e.g., the foundation will fund child health but not HIV, or will fund programs in Africa but not in Asia), and the cultural influence of funders on the overall field to which the philanthropy is being directed (e.g., emphasizing individual rather than collective responsibility, or focusing on technological interventions rather than indigenous capacity building). However, this dimension of power is also more difficult to observe, as it is indirect, informal, and may not be made explicit [41],[42]. Text S3 further describes our sources of data used to evaluate each component, including endowment disclosures with the US Internal Revenue Service and stock holdings with the US Securities and Exchange Commission (SEC).

## Comparative Analysis of Leading US Global Health Foundations

### Where Does the Money Come From?

The Bill & Melinda Gates Foundation's endowment mainly comes from Bill Gates' personal fortune and stock in Berkshire Hathaway given to the Foundation as a gift from Hathaway's CEO Warren Buffett. In 2006, Buffett made a pledge to gradually give away all of his Berkshire Hathaway stock to the Bill & Melinda Gates Foundation, most recently with an additional 24.7 million shares in July 2010 [43]. Currently, the Bill & Melinda Gates Foundation is listed with the SEC as a 10% owner of the Berkshire company [44].

At the end of 2008, the Bill & Melinda Gates Foundation Trust had US$29.6 billion assets under its management: $13.5 billion were in corporate stock, $1.8 billion in corporate bonds, $6.1 billion in US and state government obligations, and $8.2 billion in other investments, land, and temporary holdings [45].

As shown in Table 3, the Bill & Melinda Gates Foundation's corporate stock endowment is heavily invested in food and pharmaceutical companies, directly and indirectly (see Text S4 for full listings). The Foundation holds significant shares in McDonald's (9.4 million shares representing about 5% of the Gates' portfolio), and Coca-Cola (>15 million shares, over 7% of the Foundation's portfolio, not counting Berkshire Hathaway holdings). In 2009 the Bill & Melinda Gates Foundation sold extensive pharmaceutical holdings in Johnson & Johnson (2.5 million shares), Schering-Plough Corporation (14.9 million shares), Eli Lilly and Company (about 1 million shares), Merck & Co. (8.1 million shares), and Wyeth (3.7 million shares) [46].

**Table 3:** Summary of Bill & Melinda Gates Foundation stock portfolios, 2010.

Entity	Holding	Portfolio Rank	US Dollars, Billion	Portfolio Share	Company Share
**Bill & Melinda Gates Foundation Trust**	Berkshire Hathaway	1	5.89	49.75%	3.19%
	McDonald's	2	0.62	5.21%	0.88%
	Coca-Cola	4	0.51	4.31%	0.44%
	Waste Management	5	0.49	4.15%	3.25%
	Walmart	7	0.44	3.75%	0.13%
	Coca-Cola FEMSA	9	0.35	2.97%	2.47%
	Costco	10	0.34	2.83%	1.39%
	Monsanto	>20	0.02	0.19%	0.20%
	*Total*	—	11.85	—	—
**Berkshire Hathaway**	Coca Cola	1	10.0	21.58%	8.68%
	Procter & Gamble	4	4.7	10.08%	2.75%
	Kraft Foods	5	2.9	6.34%	6.03%
	Johnson & Johnson	6	2.4	5.25%	1.50%
	Walmart	7	1.9	4.04%	1.07%
	Nestle	23	0.16	0.35%	0.09%
	Sanofi-Aventis	29	0.12	0.26%	0.15%
	GlaxoSmithKline	34	0.05	0.11%	0.06%
	*Total*	—	46.44	—	—

Notes: (1) Because one-half of Gates endowment is held in Berkshire Hathaway, a figure which will rise in the future, we also report the holdings of Berkshire Hathaway. Data retrieved from http://www.sec.gov/edgar.shtml (accessed 30 June 2010). Company share calculated based on dividing number of shares held by total company shares outstanding. See Text S4 for full listing of stock holdings. (2) The largest stock-related holding of Ford Foundation is in MFO capital international emerging markets (∼$400 million) and TCW strategic mortgages (∼$490 million); of Rockefeller Foundation is in Adage Capital Partners (∼$98 million); and of W. K. Kellogg is in Kellogg stock, a producer of snack foods and cereals ($4.1 billion). Robert Wood Johnson Foundation's main stock holding reported to the SEC is in Johnson & Johnson, valued at over $800 million.

About half of the Bill & Melinda Gates Foundation's stock holdings are already invested in Berkshire Hathaway, a conglomerate holding company owning several subsidiary companies, including banks, railroads, candy production, retail, and utilities. Thus, it is necessary to examine the holdings of the Berkshire company to analyze the Foundation's stock holdings. Berkshire Hathaway's largest investment is in Coca-Cola. It owns an additional 8.7% of Coca-Cola (Warren Buffett's firm is the largest shareholder in Coca-Cola, having stock worth >$10 billion dollars) and 6.3% of Kraft (Buffett is also the largest shareholder of Kraft) [47]. Berkshire Hathaway also has significant ownership in GlaxoSmithKline, Sanofi-Aventis, Johnson & Johnson, and Procter & Gamble, and is one of the main global investors in the latter two pharmaceutical companies. Since Buffett is gradually transferring ownership of Berkshire Hathaway stock to the Bill & Melinda Gates Foundation [48], the Foundation will soon be the largest stakeholder of Coca-Cola and Kraft in the world.

Endowment investments in pharmaceutical and food companies were similarly observed among the Ford, Rockefeller, W. K. Kellogg Foundation, and Robert Wood Johnson Foundations [44],[49]–[53]. The invested companies included, to name a few, Coca-Cola, Kellogg (a leading producer of snacks and breakfast foods and the main investment of Kellogg Foundation), PepsiCo, Pfizer, GlaxoSmithKline, McDonald's, Nestle (a company with 6,000 brands mainly in food and beverage including coffee, water, chocolate, confectionery, ice cream, “health-care nutrition”, and frozen foods, among others); NovoNordisk, YumBrands (the world's largest restaurant company, operating Pizza Hut, KFC [Kentucky Fried Chicken], and Taco Bell, among others), Johnson & Johnson (main investment of Robert Wood Johnson Foundation), and Sanofi-Aventis, in addition to several mining, petrochemical, and alcohol companies. Additionally, the Ford Foundation had holdings in the tobacco company Lorillard [44], while W.K. Kellogg and Rockefeller foundations were indirectly invested in tobacco corporations through conglomerate equity funds such as Cedar Rock Capital and Adage Capital Partners, respectively. However, foundations may have guidelines to align their investment portfolio with their programmatic mission, such as the Rockefeller Foundation's “Social Investing Guidelines” [54].

Bill and Melinda Gates, according to their own statements, guide the managers of the Foundation's endowment, Cascade Investment LLC (a private investment and holding company controlled by Bill Gates holding a 20% stake in Coca-Cola FEMSA, a Coca-Cola bottling company, in 2008 and a large stake in FEMSA, the main bottling company of alcoholic and soft drinks, especially in Mexico), and regularly assess the endowment's holdings. As noted by the Foundation's description of management practices, “When instructing the investment managers, Bill and Melinda also consider other issues beyond corporate profits, including the values that drive the foundation's work. They have defined areas in which the endowment will not invest, such as companies whose profit model is centrally tied to corporate activity that they find egregious. This is why the endowment does not invest in tobacco stocks.” The Foundation had invested in Philip Morris bonds prior to a *New York Times* report on the matter in the year 2000 [28].

The Bill & Melinda Gates Foundation conflict of interest policy also sets out guidelines for employees with stockholdings [55], recognizing that “Holding a financial interest in an organization does not necessarily create a conflict of interest. It will depend upon the facts and your role as a foundation employee in selecting the entity for the proposed transaction” and, in particular, the policy states that “holding a financial interest in Berkshire Hathaway or Microsoft does not necessarily create a conflict of interest and there is no need to disclose this information”.

### Who Decides?

The Ford Foundation has a mission statement to “reduce poverty and injustice, strengthen democratic values, promote international cooperation, and advance human achievement” [56]. The Rockefeller Foundation also adopts a statement of purpose, which on its founding specified “To promote the well-being of mankind throughout the world,” and currently states “Today we apply this mission in the era of globalization” [57]. Finally, the Bill & Melinda Gates Foundation has no mission statement but has 15 guiding principles (the first is that it is “driven by the interests and passions of the Gates family”). Mr. Gates also writes an annual letter, setting out the Foundation's agenda; the most recent states that “overall we have about 30 innovations we are backing. Although the list includes only one new vaccine and one new seed, we are funding vaccines for several diseases (malaria, AIDS, tuberculosis, etc.) and new seeds for many crops (corn, rice, wheat, sorghum, etc.) …A few things we do, like disaster relief and scholarships do not fit this model, but over 90% of our work does” [58].

Like the other private foundations, the Bill & Melinda Gates Foundation does not disclose the detailed discussions that take place among its Board when funding decisions are made, because of the need to ensure free comment on grant applications. However, the Foundation's detailed policy on potential conflicts of interest for its employees [55] states that an employee has a potential conflict of interest when “He or she or any member of his or her family may receive a financial or other significant benefit as a result of the individual's position at the foundation; The individual has the opportunity to influence the foundation's granting, business, administrative, or other material decisions in a manner that leads to personal gain or advantage; or The individual has an existing or potential financial or other significant interest which impairs or might appear to impair the individual's independence in the discharge of their responsibilities to the foundation.”

The Bill & Melinda Gates Foundation's management committee oversees all the Foundation's efforts (analogous to a board of directors). The management committee comprises three co-chairs (Bill Gates, Melinda Gates, and William Gates Sr.); three trustees (Bill and Melinda Gates and Warren Buffett); a CEO (Jeff Raikes); three presidents of its respective programs in Global Development (Sylvia Mathews Burwell), United States (Allan Golston), Global Health (Tachi Yamada); chief administrative (Martha Choe), financial (Richard Henriques), human resources (Franci Phelan), and communications officers (Kate James); a managing director of public policy (Geoff Lamb); and a general counsel and secretary leading the legal team (Connie Collingsworth). Each program has a leadership team, such as the Global Health Leadership team, led by the president of the program.

Several of the Foundation's members of the management committee, leadership teams, affiliates, and major funders are currently or were previously members of the boards or executive branches of several major food and pharmaceutical companies (see the commercial network of the Bill & Melinda Gates Foundation in the interactive map tool at http://mapper.nndb.com/start/?map=12051; see also Figure S1 for more details), including Coca-Cola, Merck, Novartis, General Mills, Kraft, and Unilever (one of the largest global consumer goods companies owning product brands in food, beverage, and personal and home care), among others [59]–[62].

Given the important role of these board members in shaping and approving grant allocation decisions as well as the Foundation's strategic vision, the personal histories of leading members of the board were analyzed. The Foundation is described as “led by CEO Jeff Raikes and Co-chair William H. Gates Sr., under the direction of Bill and Melinda Gates and Warren Buffett.” Warren Buffett, the second-largest donor to the Foundation and a board member, was a member of the board of Coca-Cola from 1989–2006 (Figure S1; his son, Howard Buffett, is on the board of Coca-Cola Enterprises and ConAgra Foods [one of North America's largest packaged food companies], which is invested in modern seed technology, as are other members of the board of Berkshire Hathaway). Warren Buffett is the chairman and CEO of Berkshire Hathaway (and Bill Gates is a director on its board). Jeff Raikes, who replaced Patty Stonesifer in May 2008 (currently a senior advisor to the trustees), retired as the president of Microsoft business division to join the Foundation. Both Raikes and William Gates Sr. are on the board of Costco Wholesale Corporation (a membership warehouse club, selling bulk-packaged products at very high volume and low prices—the third largest retailer in the US).

Further overlaps between Bill & Melinda Gates Foundation leadership, other private foundations, and circular flows of personnel with food and pharmaceutical companies were observed (Text S5). Such patterns of interlinked board directorships, common among corporations and nonprofit organizations, were similarly found in the other private foundations studied.

The Gates Foundation conflict of interest policy (as mentioned above) [55] notes that in such cases of overlap, the employee should disclose his/her position; refrain from exercising decision-making authority with respect to the grant and also with helping the potential grantee with preparing or submitting proposals; and recuse oneself from managing the grant. Further, it notes that board members of other organizations have fiduciary duties of care, loyalty, and confidentiality to those organizations, which require directors to act in accordance with the best interest of the organization on whose board the director serves, irrespective of the other entities with which the director is affiliated. Where recusal is not possible, the policy states that “you should document that your manager at the foundation has approved any final decisions with respect to the foundation's grant to the organization.”

### Who Benefits? Where Does the Money Go?

The bulk of the Bill & Melinda Gates Foundation's financial transfers in global health have been to programs developing medical technologies [24]: more than 97% of its financial disbursements are directed to infectious diseases, and less than 3% to chronic noncommunicable diseases [16]. The majority of the Foundation's grants are directed to researchers in the US. Of US$9 billion in financial transactions that went toward 1,094 global health programs between 1998 and 2007, about half went to recipients in the US and 40% to supranational organisations; overall, about 42% of all funding was spent on health care delivery or increasing access to drugs, vaccines, and medical commodities, while an additional one-third was allocated to technology development (mainly for vaccines and microbicides) or basic science research [24].

Several grants are linked to companies that are represented on the Foundation's board among its investments. The Foundation has established partnerships with the Coca-Cola Company, which, in the words of the Foundation, are intended to “create new market opportunities for local farmers whose fruit will be used for Coca-Cola's locally-produced and sold fruit juices” [63]. The program is a four-year, $11.5 million partnership for “mango and passion fruit farmers to participate in Coca-Cola's supply chain for the first time,” with a $7.5 million grant provided by the Bill & Melinda Gates Foundation to TechnoServe, $3 million provided by The Coca-Cola Company, and $1 million by bottling partner Coca-Cola Sabco. This could reflect the recent comments of Melinda Gates in a webcast, “What we can learn from Coke,” suggesting that government agencies and nongovernmental organizations (NGOs) could learn from the manufacturer to promote global health in low- and middle-income countries [64].

In addition, many of the Foundation's pharmaceutical development grants may benefit leading pharmaceutical companies such as Merck and GlaxoSmithKline [24], for example via partnerships to test pneumonia and rotavirus vaccines (such as the ROTATEQ partnership and the Merck Vaccines network partnership with the Global Alliance for Vaccines and Immunizations network), experimental malaria vaccines (through Medicines for Malaria Venture, an NGO), cervical cancer vaccines (through PATH, an NGO, and Merck's vaccine Gardasil), and HIV interventions (through the Africa Comprehensive HIV/AIDS partnership). Johnson & Johnson has entered a clinical partnership to develop new HIV-prevention technology, noting “the work between Johnson & Johnson and the Gates Foundation is a strong, strategic, comprehensive relationship” [65].

The Foundation has also funded initiatives that are not obviously linked to corporations, such as those for health surveillance and monitoring of disease burdens. It invested $105 million to create the Institute for Health Metrics and Evaluation at the University of Washington, which is led by one of the leading researchers responsible for the Global Burden of Disease Study (one of the most widely used datasets in global health) and has now become a leading institution for estimating global mortality and disability rates. The Foundation is also one of the largest donors to the WHO, giving sums that amount to about 4% of WHO's overall budget (∼$150 million) [17].

## Discussion

Here, we have observed that five major US private nonprofit foundations have significant investments in food and/or pharmaceutical companies, directors who currently or have previously sat on the boards of those companies, and, in several cases, enter in partnerships with those companies. Our analysis focused mainly on the potential conflicts of interests among the largest foundation, the Bill & Melinda Gates Foundation. As one example, we found that Bill & Melinda Gates Foundation has substantial holdings in the Coca-Cola Corporation, and also participates in grants that encourage communities in developing countries to become business affiliates of Coca-Cola. It has been noted by some commentators that sugary drinks such as those produced by Coca-Cola are correlated with the rapid increase in obesity and diabetes in developing countries [66]. Noncommunicable diseases constitute more than half of all deaths in low- and middle-income countries [67], while the Bill & Melinda Gates Foundation, along with the World Bank and national official development aid donors, have been criticized for directing less than 3% of their collective budgets toward these conditions [16],[24],[68]. Similar potential conflicts of interests were observed among other leading global health nonprofit foundations, including the Ford Foundation, Rockefeller Foundation, Robert Wood Johnson Foundation, and W.K. Kellogg Foundation.

Before assessing the implications of these findings, important limitations of our study must be addressed. First, as with all studies into the sociology of institutions [37],[39], we can observe the flows of funding and interlocking members of different institutions, but we cannot assess whether these associations resulted in a particular decision taking place. At a minimum, our observations indicate that some of the same people who participate on the boards of major multinational pharmaceutical and food corporations are also linked to the managerial boards of the Bill & Melinda Gates Foundation. Second, the situation changes rapidly as, in the case of investment holdings, the information provided is already dated at the time of this communication given daily changes in stock portfolios. Third, mapping the institutional relationships between representatives of boards of directors and their other affiliations is a complex task and, to our knowledge, no standardized dataset exists for this purpose. Thus, it is possible that the links we demonstrate in Figure S1 are not exhaustive and that several links we report pre-date the year 2008. Fourth, we have focused our attention on private foundations given the dearth of prior academic analyses in this realm. However, nonprofit organizations and universities have also been accused of bias against the private sector, with ideologically driven critiques and financial connections that favor government-based bureaucracies (although corporate leaders sit on the boards of major US universities) [69]. Similarly, influence is not unidirectional from corporations to nonprofit bodies; the latter can influence corporations in the many modes of “corporate philanthropy” and social responsibility. Hence, our focus of analysis on private institutions does not provide a comprehensive review of the types of interactions and interests that can exist in the global health sphere. Finally, our analysis has also not addressed the potential for conflicts of interest to arise with the Bill & Melinda Gates Foundation's grants related to the agricultural sector and investments in seed technologies, pesticides, and genetically modified organisms in connection with Monsanto (in which the Foundation holds stock and with which it supports joint grants through the African Agricultural Technology Foundation that may benefit the company) as well as the Foundation's stock holdings in oil companies, such as ExxonMobil and British Petroleum, that have been linked to environmental and health crises in the Niger Delta and other regions [70].

A private foundation clearly has the legal right to spend money however it wishes within the limits of the law; yet, in an environment where private foundations influence the future direction of, for example, what programs will be introduced into a foreign community, in a manner that does not necessarily involve directorship or voting from the community members themselves, it is reasonable to subject the decision-making processes of these entities to public debate, especially if these funds were to have otherwise been collected for public redistribution through federal taxation.

Several strategies may help mitigate the potential for conflicts of interests in private foundations to negatively impact global health decision-making (also Box 1):

**Box 1. Strategies for Mitigating Conflicts of Interest**

1. **Divestment:** Investments should not counteract the foundation's charitable mission statement and objectives in either a real or a perceived way. There should be separation between investment management and the foundation's board. It is also possible to create a “blind trust” so that the foundation leaders are not aware of their corporate investments and avoids the possibility that investment knowledge influences program decisions.

2. **Transparency:** Full disclosure of actual or potential conflicts of interest is necessary in making public health decisions. This includes disclosing corporate affiliations (directorships, advisory panels, funding receipt) and personal investment holdings. When board members and their friends and relatives stand to be directly affected by grant decisions, they should recuse themselves from the discussion and decision. Members may also resign from corporate boards and sell stock (or create a blind trust, as described above). Independent audits can also routinely monitor the scope and management of potential conflicts of interest within private foundations. A widely shared and monitored public health code of ethics could provide guidance to mitigate conflicts of interest at foundations from adversely influencing practice.

3. **Alignment of aid with community needs:** Foundation investment and program portfolios should incorporate representation from the intended recipients of its support.

### (1) Divestment

Private foundations have been advised to not invest in companies that stand to profit from the tax-exempt foundation's agenda or in those that produce products such as tobacco, salty foods, or sugary drinks that have well-established connections to global health epidemics [71]. Such investments may counter these foundations' purposes of promoting global health. Similarly, foundations must be wary about investing in pharmaceutical and food and beverage companies that could gain market share and enhanced publicity, and could benefit as a result of the foundation's grants. For example, the Bill & Melinda Gates Foundation held stock in Merck at a time when it developed partnerships with the African Comprehensive AIDS and Malaria Partnership and the Merck Company Foundation to test Merck products. As another instance, which may reflect aligning interests, the Robert Wood Johnson Foundation has played a leading role in promoting anti-tobacco products and maintains Smoking Cessation Leadership Centers and programs [72], although its endowment is mainly invested in Johnson & Johnson, a leading manufacturer of cessation products, and some board members have been represented on both the Foundation's and the company's boards [53].

### (2) Transparency

Given that there is a limited pool of specialist expertise that can be drawn upon, it is possible that situations may arise where some persons who sit on the boards of, or advise, philanthropic foundations, will also have links to corporations. It would be unreasonable to demand that foundations refuse to engage with those who have corporate links, so denying themselves the best advice in some fields. Many authors have stated that a solution to this issue is to adopt full disclosure, or transparency, and to ensure that all individuals on foundation boards recuse themselves from decisions related to their affiliate companies [16]. However, achieving transparency and appropriate recusals is not always easy. Indeed, while the information we collected to conduct this case study is all in the public domain (and thus transparent), it required considerable time and effort to assemble in a manner that can be publicly interpreted, thus limiting full transparency. Furthermore, there is an inevitable time lag between decisions being made and information on the relationships among those making the decisions becoming available, which again limits transparency. Nevertheless this case study sets out the type of information that is required for observers to interpret global health financial flows and, we argue, offers a template for future disclosures to enhance transparency of not just individual employees but the full scope of potential conflicts of entire institutions.

### (3) Aligning Aid with Community Needs

It is important to align aid with community needs. However, despite numerous declarations to align aid with the preventable burden of disease, private foundations tend to operate in “silos,” focused on a core set of issues that their founder or governing director decides is important [73]. Extra-budgetary contributions, earmarked by donors, are a major factor in creating the observed imbalance between patterns of expenditure by global health institutions and the current burden of disease [17].

Major funders can have a significant impact on overall financial flows; International Monetary Fund (IMF) economists are advising that countries divert aid to reserves because of concerns about its unreliable and unpredictable nature, and the desire to privatize health care services rather than perpetuate state-run health care operations [74],[75]. A recent study found that when countries are borrowing from the IMF more than 98% of aid is diverted to reserves or displacing government spending on health [75]. Working toward global health financing that aligns with the disease burden will remain elusive if foundations and financial institutions operate as distinct bodies while influencing communities that are receiving numerous disparate and poorly coordinated funds. Thus, foundation investment and program portfolios should incorporate representation from the intended recipients of its support.

## Conclusion

The question of whether and how financial and institutional relationships might shape foundation decision-making has yet to be answered, and continues to be a point of controversy. To draw a recent example, if a Chinese businessperson invested in the Chinese auto industry were to donate to the Hurricane Katrina relief efforts in New Orleans, such a donation would no doubt be welcomed; if the donation, however, was conditional upon renovating New Orleans′ factories to produce products for the Chinese auto industry, such a program may produce legitimate debate. Analogously, when the Gates Foundation is heavily invested in Coca-Cola and simultaneously works to orient developing country farmers towards production for Coca-Cola instead of alternative development strategies, such an approach has potential consequences for grant-receiving communities and their health [21],[76]–[79].

Potential conflicts of interest exist everywhere, but there is considerable variation in how they are managed. When such topics have been discussed in the medical literature in the past, they have resulted in the avoidance of disastrous outcomes—such as the revelation that major biomedical research was inappropriately analyzed to support drug marketing by academics influenced by the pharmaceutical industry [80]. The overarching challenge is to prevent these potential interests from distorting science and public health outcomes.

## Supporting Information

Figure S1 Sampling of major interlinkages of leading members of the Gates Foundation Board of Directors and advisory panels. File provides access to externally hosted information.
(DOC)

Text S1 Historical controversy about the politics of philanthropy.
(DOC)

Text S2 Rules and regulations governing US private foundations.
(DOC)

Text S3 Sources of data.
(DOC)

Text S4 Full corporate stock investment listings filed with the SEC (Form 13-HR), Bill & Melinda Gates Foundation Trust and Berkshire Hathaway Holdings, 30 June 2010.
(DOC)

Text S5 Examples of interlocking appointments with food, pharmaceutical companies, and other private foundations.
(DOC)

## Abbreviations

CEO: chief executive officer
NGO: nongovernmental organization
RBM: Roll Back Malaria
SEC: US Securities and Exchange Commission
WHO: World Health Organization
