# Supplementary material for: Global Health Philanthropy and Institutional Relationships: How Should Conflicts of Interest Be Addressed?
Source: PLoS Med. 2011 Apr 12;8(4):e1001020. doi: 10.1371/journal.pmed.1001020 (PMC3075225; doi:10.1371/journal.pmed.1001020)
Supplement: Figure S1 — Sampling of major interlinkages of leading members of the Gates Foundation Board of Directors and advisory panels. File provides access to externally hosted information. (DOC) [file pmed.1001020.s001.doc]

**Supporting Information Figure 1 Sampling of Major Interlinkages of leading members of the Gates Foundation Board of Directors and advisory panels**

Web Link: <http://mapper.nndb.com/start/?map=12051> and

Summary: <http://mapper.nndb.com/maps/087/000012051/>

*Notes:* Latest available data covering links prior to May 2008 retrieved from SEC Edgar database and mapped using NNDB (accessed July 10^th^, 2010).
