# Supplementary material for: Global Health Philanthropy and Institutional Relationships: How Should Conflicts of Interest Be Addressed?
Source: PLoS Med. 2011 Apr 12;8(4):e1001020. doi: 10.1371/journal.pmed.1001020 (PMC3075225; doi:10.1371/journal.pmed.1001020)
Supplement: Text S1 — Historical controversy about the politics of philanthropy. (DOC) [file pmed.1001020.s002.doc]

**Supporting Information Text S1. Historical controversy about the politics of philanthropy**

Controversies about the politics and philosophies of private foundations are not new. Between 1880 and 1930,[1] so-called ‘robber barons’ were criticized for starting tax-exempt foundations to relieve political pressure for the greater financial redistribution in American society [2]. For example, the Rockefeller Foundation grew out of the Standard Oil Company and was criticised for supporting initiatives seen as preventing the unionization of workers [3]. The American Walsh Commission on industrial relations suggested that Mr. Rockefeller pay workers higher salaries[4], stating that his foundation “appear[s] to be a menace to the welfare of society” by preventing the taxation of the family’s fortune and thereby reducing government funding for welfare programs during periods of recession. The Foundation previously promoted a free-market ideology emphasising (in the words of its president) that “The solution to the problems of ill health in modern American society involves individual responsibility” [5]. Hence, the eradication of disease was promoted through behavioural change, educational programs and technological interventions rather than the economic redistribution of wealth or strengthened social safety-nets [6]. The Foundation was later criticized for becoming a leading funder of the eugenics movement[7], and subsequently for population control programmes linked to President Nixon’s declaration that population growth in developing countries reduced access to key raw materials and was therefore a “national security threat”[8]. More recently, the Foundation was criticised for having advanced the entry of genetically-modified crops into new markets against popular opposition, while having financial investments in genetically-modified-seed-producing companies [9].

Summarising the prevailing sociological critique of the time, two commentators argued that “money which ought to be in the hands of the public is being retained by aristocrats for purposes beyond the control of democratic institutions; the academic freedom of universities is being subverted by control of academic budgets by the foundations; public policy is being determined by private groups; the scientific and scholarly research and the artistic creativity of individuals are being stifled by the emphasis of foundations on group-research; smallness and individual effort are thwarted by materialistic and business-oriented demands of foundation management; foundations are bastions of an elite of white, Anglo-Saxon, Protestant managers holding out against the normal development of a pluralistic and ethnic society” [2].
