# Supplementary material for: Global Health Philanthropy and Institutional Relationships: How Should Conflicts of Interest Be Addressed?
Source: PLoS Med. 2011 Apr 12;8(4):e1001020. doi: 10.1371/journal.pmed.1001020 (PMC3075225; doi:10.1371/journal.pmed.1001020)
Supplement: Text S2 — Rules and regulations governing US private foundations. (DOC) [file pmed.1001020.s003.doc]

**Supporting Information Text S2. Rules and Regulations Governing U.S. Private Foundations**

The main defining feature of a foundation, whether a private foundation or public charity, is that they are not for profit and qualify for a federal tax exemption according to 26 U.S.C. § 501(c)(3) of the Internal Revenue Service code. The U.S. common law system and tax codes of the Internal Revenue Service provide federal tax exemptions to an organization if it is organized exclusively for one of the following exempt purposes: religious, charitable, scientific, testing for public safety, literary, educational purposes, to foster national or international amateur sports competition, promote the arts, or for the prevention of cruelty to children or animals [1].

There are two types of U.S. federal tax-exempt organizations: public charities and private foundations. An organization is assumed to be a private foundation unless it meets criteria for a public charity. Public charities, such as the American Cancer Society, usually derive their funding from the general public. One important distinction is that public charities may directly provide services and deliver care, whereas private foundations’ operations are limit to making grants to other agencies that act on the foundations’ behalf (and thus they are sometimes called ‘non-operating foundations’). Further rules apply at the state level to the governance of private foundations that may exempt them from state taxation.

Federal tax exemption requires that the foundations must “not be organized or operate for the benefit of private interests, such as the creator or creator’s family, shareholders of the organization, other designated individuals, or persons controlled directly or indirectly by such private interests”[2]; no private shareholder or individual may inure from the organization’s net earnings; and there are restrictions on their political and lobbying activities (see IRS 501(c)(3) for more details).

To prevent conflicts of interests, foundations often have explicit policies. These usually set out ethical criteria for their staff, directors, and recipients of grants. The Ford Foundation, for example, has documents establishing ‘standards of independence’, a ‘trustee code of ethics’, guidelines for ‘ensuring grants are used for charitable purposes’, ‘procedures for approving affiliated grants’, ‘procedures for the receipt, retention, and treatment of complaints’ and a ‘staff code of conduct and ethics.’

It is also important to bear in mind that some organizations use the title ‘foundation’, although the designation does not have legal status (which can only be ascertained by examining whether there private foundation has filed a 990-PF with the IRS). More than 120,617 private foundations were estimated to exist in 2009 and 88,879 filed with the IRS in 2010 [3]. Less than 5% of these private foundations were operating foundations. Private foundations are estimated to manage more than $560 billion in assets and contribute more than $44 billion each year.

Foundations are designed to fulfil the missions of their donors. Thus, donors can also push foundations to adopt particular philosophies or orientations that are favourable to their interests. This was observed in the sociological programmes of tobacco companies aiming to promote a particular market-oriented, technological, and anti-government philosophy based on concepts of individual responsibility and personal freedom, which would influence public beliefs and create favourable social conditions for selling tobacco [4].

There are multiple benefits to establishing a private foundation. One is the avoidance of liability for the consequences of the foundation’s activities by the parent corporation or donor (using the Hilton Foundation as an example, the legal designation of a private foundation includes disclaimers such as “Although both organisations carry out charitable activities and have Hilton in their name, we [the Hiltons] are not affiliated in any way”). Another is tax-exemption. Donors receive an income tax deduction and an unlimited estate tax deduction for bequests at death. However, these donations effectively reduce the government’s tax income, and thus justify public scrutiny because money that goes untaxed from private funders is supposed to be used for the public benefit, being displaced from alternative public spending programs. Some major U.S. donors have decried the tax situation where billionaires can pay less tax than their secretaries[5]. A third benefit, albeit difficult to quantify, is the positive social reputation and feelings of goodwill that accrue to the foundation’s creators.
