# Supplementary material for: Global Health Philanthropy and Institutional Relationships: How Should Conflicts of Interest Be Addressed?
Source: PLoS Med. 2011 Apr 12;8(4):e1001020. doi: 10.1371/journal.pmed.1001020 (PMC3075225; doi:10.1371/journal.pmed.1001020)
Supplement: Text S3 — Sources of data. (DOC) [file pmed.1001020.s004.doc]

**Supporting Information Text S3. Sources of Data**

First, to address the sources of funding, data were taken from financial and policy statements from private foundations and their managerial boards [1], as well as investment reports and endowment disclosures to the Securities and Exchange Commission (SEC) Trading Investment Portfolio in its Edgar database [2] and filings with the IRS for the 2008 calendar year [3]. It was necessary to interrogate market data to evaluate the specific stock investments of the Foundation, as these purchases are also not immediately publicly disclosed; there is often a delay of about 4-5 months in tracking them. Second, as with most Foundations, the decisions about grant-making allocations and priorities are made by the board of directors. Data on the board membership were found on the foundations’ websites. Unfortunately, there is no routinely available dataset on institutional linkages of board members to corporations, universities, and political organizations. Thus, these ties were tracked using a publicly available network mapping software package, NNDB, which provides a map of current and historical linkages of affiliated members of the Foundations [4]. To assess the validity of this data, a random sample of 20% of the linkages were investigated further using additional data from the SEC EDGAR database, and found to be accurate prior to 2008. This analysis was further supplemented with a biographical examination of board members. Lastly, the question of who benefits from these decisions is a challenging empirical question, as the methods of determining impact on intended recipients of aid are subject to considerable debate. We narrowed the question to what subject matter specific grants were directed towards (e.g., malaria, tuberculosis) from the Foundation and which organizations derived won grants, in the Foundation’s words and reports.

P990 Forms containing full data on government obligations, corporate bonds, corporate stock, and other investments (including land) can be found on the website of the National Center for Charitable Statistics: available at http://nccsdataweb.urban.org/PubApps/990search.php/ (Accessed January 7^th^, 2011).

Securities and Exchange Commission. Edgar Database. 2010. Available at: http://www.sec.gov/cgi-bin/browse-edgar?action=getcompany&CIK=0001166559&owner=exclude&count=40
