# Supplementary material for: Global Health Philanthropy and Institutional Relationships: How Should Conflicts of Interest Be Addressed?
Source: PLoS Med. 2011 Apr 12;8(4):e1001020. doi: 10.1371/journal.pmed.1001020 (PMC3075225; doi:10.1371/journal.pmed.1001020)
Supplement: Text S4 — Full corporate stock investment listings filed with the SEC (Form 13-HR), Bill & Melinda Gates Foundation Trust and Berkshire Hathaway Holdings, 30 June 2010. (DOC) [file pmed.1001020.s005.doc]

**Supporting Information Text S4. Full Corporate Stock Investment Listings filed with the Securities and Exchange Commission (Form 13-HR), Bill & Melinda Gates Foundation Trust and Berkshire Hathaway Holdings, June 30^th^, 2010**

| **Bill & Melinda Gates Foundation Trust Corporate Stock Investments** | | |
| --- | --- | --- |
| Company Stock | Value ($1,000) | Portfolio Share (%) |
| Berkshire Hathaway | 5,896,853 | 49.75 |
| McDonald’s | 617,367 | 5.21 |
| Caterpillar Inc | 576,095 | 4.86 |
| Coca-Cola Co | 510,322 | 4.31 |
| Waste Management | 491,765 | 4.15 |
| Canadian National Railway | 481,972 | 4.07 |
| Walmart | 444,792 | 3.75 |
| Exxon Mobil Corp | 407,700 | 3.44 |
| Coca Cola FEMSA | 351,541 | 2.97 |
| CostCo | 335,998 | 2.83 |
| Grupo Televisa | 271,703 | 2.29 |
| Autonation Inc | 210,446 | 1.78 |
| British Petroleum | 206,001 | 1.74 |
| Crown Castle Intl | 198,704 | 1.68 |
| FedEx | 177,028 | 1.49 |
| Ecolab Inc. | 112,275 | 0.95 |
| America Movil | 94,999 | 0.80 |
| CSX Corporation | 79,408 | 0.67 |
| Goldman Sachs Group | 65,635 | 0.55 |
| M & T Bank Corp | 63,712 | 0.54 |
| Expenditors Intl Wash | 56,873 | 0.48 |
| Republic Services Inc | 40,136 | 0.34 |
| Expedia Inc | 28,170 | 0.24 |
| Eastman Kodak Co | 24,955 | 0.21 |
| Devon Energy Corp | 24,825 | 0.21 |
| Cabot Oil & Gas Corp | 24,290 | 0.20 |
| Monsanto | 23,110 | 0.19 |
| Range Resources | 23,086 | 0.19 |
| IAC Interactive Corp | 11,754 | 0.10 |
| Greater China Fund | 1,806 | 0.02 |
| Bank of Florida | 24 | 0.00 |
| Total | $11,853,345 |  |

Source: Securities and Exchange Commission. Edgar Database. 2010. Available at: http://www.sec.gov/cgi-bin/browse-edgar?action=getcompany&CIK=0001166559&owner=exclude&count=40

| **Berkshire Hathaway** | | |
| --- | --- | --- |
| Company Holdings | Value ($1,000) | Portfolio Share (%) |
| Coca Cola | 10,024,000 | 21.58 |
| Wells Fargo & Co | 8,194,263 | 17.64 |
| American Express Co. | 6,018,944 | 12.96 |
| Procter & Gamble Co. | 4,682,699 | 10.08 |
| Kraft Foods Inc | 2,946,008 | 6.34 |
| Johnson & Johnson | 2,440,334 | 5.25 |
| Wal-Mart Stores, Inc | 1,876,515 | 4.04 |
| Wesco Finl Corp | 1,843,238 | 3.97 |
| US Bancorp | 1,543,031 | 3.32 |
| ConocoPhillips | 1,428,992 | 3.08 |
| Washington Post Co. | 709214 | 1.53 |
| Moody's | 613,214 | 1.32 |
| Nike Inc. | 516,150 | 1.11 |
| M&T Bank Corporation | 455,657 | 0.98 |
| Republic Services Inc. | 321,908 | 0.69 |
| CostCoWholesale Corp | 237,598 | 0.51 |
| USG Corporation | 206,232 | 0.44 |
| Fiserv Inc. | 200,904 | 0.43 |
| Comcast Corp | 197,160 | 0.42 |
| Ingersoll-Rd Company LTD | 194,386 | 0.42 |
| Nalco Holding Co. | 187,209 | 0.40 |
| Iron Mountain Inc. | 179,680 | 0.39 |
| Nestle | 164,585 | 0.35 |
| Carmax Inc. | 153,745 | 0.33 |
| Torchmark Corp | 139811 | 0.30 |
| Lowes Companies Inc | 132,730 | 0.29 |
| Becton Dickinson & Co. | 127,794 | 0.28 |
| NRG Energy Inc | 127,260 | 0.27 |
| Sanofi Aventis | 122,154 | 0.26 |
| General Electric Co. | 112,157 | 0.24 |
| United Parcel Service Inc | 81307 | 0.18 |
| Home Depot Inc | 77,414 | 0.17 |
| Bank of America Corp. | 71,850 | 0.15 |
| GlaxoSmithKline | 51,372 | 0.11 |
| Exxon Mobil Corp | 24072 | 0.05 |
| Gannett Inc. | 23,424 | 0.05 |
| Comdisco Holding Co. | 13,799 | 0.03 |
| Total | $46,440,810 |  |

Source: Securities and Exchange Commission. Edgar Database. 2010. Available at: http://www.sec.gov/Archives/edgar/data/1067983/000095012310078001/v56978ae13fvhr.txt

P990 Forms containing full data on government obligations, corporate bonds, corporate stock, and other investments (including land) can be found on the website of the National Center for Charitable Statistics: available at http://nccsdataweb.urban.org/PubApps/990search.php/ (Accessed January 7^th^, 2011).
