# Supplementary material for: Global Health Philanthropy and Institutional Relationships: How Should Conflicts of Interest Be Addressed?
Source: PLoS Med. 2011 Apr 12;8(4):e1001020. doi: 10.1371/journal.pmed.1001020 (PMC3075225; doi:10.1371/journal.pmed.1001020)
Supplement: Text S5 — Examples of interlocking appointments with food, pharmaceutical companies, and other private foundations. (DOC) [file pmed.1001020.s006.doc]

**Supporting Information Text S5. Examples of Interlocking Appointments with Food, Pharmaceutical Companies and Other Private Foundations**

Anne Fudge, the chairman of the Gates Foundation’s US Program Advisory Panel is also on the board of directors of Rockefeller Foundation (in addition to General Electric, Novartis, Unilever, and Harvard University, among others)[1]. She was a marketing director at General Mills and then rose to the presidency of the Beverage, Desserts and Post Division at Kraft General Foods (1986-2003), She then became CEO and chairman of Young and Rubicam Brands and Advertising (2003-2006), a “commercial communications network of preeminent companies in advertising, public relations, identity and design, sales promotion, and direct marketing.” Former director of HIV, TB, and reproductive health, Helene Gayle, is a trustee of the Rockefeller Foundation, CEO of CARE USA, and on the board of directors of Colgate-Palmolive Company, among many other institutions [2].

Members of personnel also move between the Foundation and pharmaceutical companies. For example, in April 2010, a former Merck senior vice president, Richard Henriques, became the chief financial officer of the Gates Foundation [3]. At least two other members of the Gates Foundation leadership have transferred from the leadership of GlaxoSmithKline to sit on the Foundation’s board of directors, including Kate James [4], the chief communications officer, and Tachi Yamada, until February 2011, the head of the Foundation’s global health program [5]. Similar patterns were observed with the other foundations studied.
